# Mechanism of Procedural Stroke Following Carotid Endarterectomy or Carotid Artery Stenting Within the International Carotid Stenting Study (ICSS) Randomised Trial

**DOI:** 10.1016/j.ejvs.2015.05.017

**Published:** 2015-09

**Authors:** A. Huibers, D. Calvet, F. Kennedy, K.R. Czuriga-Kovács, R.L. Featherstone, F.L. Moll, M.M. Brown, T. Richards, G.J. de Borst

**Affiliations:** a Department of Brain Repair and Rehabilitation, Institute of Neurology, University College London, London, UK; b Department of Vascular Surgery, University Medical Centre Utrecht, The Netherlands; c Centre de Psychiatrie et Neurosciences, INSERM UMR 894, Paris Descartes University, Paris, France; d Department of Neurology, Centre hospitalier Sainte-Anne, Paris, France; e Department of Neurology, Clinical Center, University of Debrecen, Debrecen, Hungary; f Department of Surgical and Interventional Sciences, University College London, London, UK

**Keywords:** Carotid artery stenting, Carotid endarterectomy, Carotid stenosis, Procedural stroke, Stroke mechanism

## Abstract

**Objective:**

To decrease the procedural risk of carotid revascularisation it is crucial to understand the mechanisms of procedural stroke. This study analysed the features of procedural strokes associated with carotid artery stenting (CAS) and carotid endarterectomy (CEA) within the International Carotid Stenting Study (ICSS) to identify the underlying pathophysiological mechanism.

**Materials and methods:**

Patients with recently symptomatic carotid stenosis (1,713) were randomly allocated to CAS or CEA. Procedural strokes were classified by type (ischaemic or haemorrhagic), time of onset (intraprocedural or after the procedure), side (ipsilateral or contralateral), severity (disabling or non-disabling), and patency of the treated artery. Only patients in whom the allocated treatment was initiated were included. The most likely pathophysiological mechanism was determined using the following classification system: (1) carotid-embolic, (2) haemodynamic, (3) thrombosis or occlusion of the revascularised carotid artery, (4) hyperperfusion, (5) cardio-embolic, (6) multiple, and (7) undetermined.

**Results:**

Procedural stroke occurred within 30 days of revascularisation in 85 patients (CAS 58 out of 791 and CEA 27 out of 819). Strokes were predominately ischaemic (77; 56 CAS and 21 CEA), after the procedure (57; 37 CAS and 20 CEA), ipsilateral to the treated artery (77; 52 CAS and 25 CEA), and non-disabling (47; 36 CAS and 11 CEA). Mechanisms of stroke were carotid-embolic (14; 10 CAS and 4 CEA), haemodynamic (20; 15 CAS and 5 CEA), thrombosis or occlusion of the carotid artery (15; 11 CAS and 4 CEA), hyperperfusion (9; 3 CAS and 6 CEA), cardio-embolic (5; 2 CAS and 3 CEA) and multiple causes (3; 3 CAS). In 19 patients (14 CAS and 5 CEA) the cause of stroke remained undetermined.

**Conclusion:**

Although the mechanism of procedural stroke in both CAS and CEA is diverse, haemodynamic disturbance is an important mechanism. Careful attention to blood pressure control could lower the incidence of procedural stroke.

**What this paper adds**

Stroke is a complication of carotid revascularisation that limits the benefit of the procedure in overall stroke prevention. To decrease the risk of revascularisation it is important to understand the mechanism of stroke. In a recent randomised trial in which patients were treated with carotid artery stenting (CAS) or carotid endarterectomy (CEA), one-third of the procedural strokes were caused by periprocedural haemodynamic disturbances. This suggests that careful attention to blood pressure control could lower the incidence of procedural stroke.

## Introduction

Stroke is a feared complication of carotid revascularisation that limits the benefit of the procedure in overall stroke prevention. Large randomised clinical trials have shown that patients treated with carotid artery stenting (CAS) have a higher risk of stroke within 30 days of intervention than patients treated with carotid endarterectomy (CEA).^1–4^ This difference in procedural stroke is mostly attributed to an excess of minor non-disabling strokes. In these recent trials^1,4^ the operative risk of CEA was significantly lower than the 7% risk of stroke or death within 30 days of treatment reported in the North American Symptomatic Carotid Endarterectomy Trial (NASCET) and the European Carotid Surgery Trial (ECST).^5,6^ In contrast, mid- and long-term results of these randomised trials show that the risk of stroke beyond the operative period is similar between both treatment arms.^1–4,7,8^ As a consequence, improvement in surgical and stenting techniques to further decrease procedural stroke risks would clearly increase the absolute benefit of carotid revascularisation. The main suggested causes of stroke related to carotid endarterectomy are embolisation, intraoperative hypoperfusion, thrombotic occlusion of the ipsilateral or contralateral carotid artery, or hyperperfusion syndrome.^9,10^ The International Carotid Stenting Study (ICSS) was a large randomised controlled clinical trial that compared CAS and CEA in recently symptomatic patients.^1^ Within ICSS, this study assessed the clinical characteristics of the procedural strokes to better understand the nature and mechanism of these adverse events related to the revascularisation procedure.

## Methods

### Patient selection

The methods of ICSS (ISRCTN 25337470) have been described previously.^1,11^ In summary, patients over 40 years old with recently symptomatic moderate or severe carotid stenosis (≥50% reduction of the lumen diameter), who were considered equally suitable for either procedure, were randomly allocated to undergo treatment by stenting or endarterectomy. Patients were excluded if the stenosis was considered unsuitable for stenting because of specific vascular anatomy, if the stenosis was caused by non-atherosclerotic disease, or if the symptomatic artery had previously been revascularised. ICSS was approved by local ethics committees for non-UK centres and by the Northwest Multicentre Research Committee in the UK.

### Outcome events

In ICSS major outcome events were adjudicated by an independent endpoint committee that was unaware of treatment allocations. Stroke was defined as a rapidly developing clinical syndrome of focal disturbance of cerebral function lasting more than 24 hours or leading to death with no apparent cause other than that of vascular origin. Stroke was considered procedural if the event occurred at any time between initiation of the revascularisation (day 0) and day 30 after revascularisation. Stroke was classified as disabling if there was an increase in the modified Rankin score to 3 or more, attributable to the event 30 days after onset.

**Figure 1:**
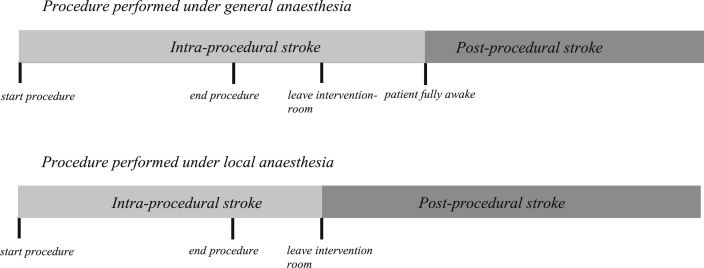
Definition of intraprocedural versus postprocedural day 0 strokes.

For the present analysis, the technical data forms completed by the surgeon or interventionist at the time of the procedure were reviewed along with the carotid and brain imaging to determine the most likely mechanism of stroke. The technical forms recorded the techniques used for endarterectomy or stenting and also recorded complications occurring during the procedure or immediately afterwards, including complications such as hypotension or asystole. In addition, the available clinical data describing the stroke together with carotid and brain imaging were interrogated. For procedures performed with the patient under general anaesthesia, stroke was considered intraprocedural if the cerebral deficit presented before the patient fully emerged from anaesthesia. Stroke was determined to have occurred after the procedure if the onset of the stroke occurred after full and asymptomatic awakening from anaesthesia including a clear symptom-free interval between the intervention and onset of symptoms. For procedures performed under local anaesthesia, stroke was considered intraprocedural if the cerebral deficit was noticed in the intervention room during the procedure and determined to have occurred after the procedure if the patient left the intervention room before the deficit was noted (Fig. 1). If stroke occurred on the day of the procedure and it was not possible to make the distinction between intraprocedural or postprocedural, timing of stroke was classified as undetermined.

### Classification of stroke mechanism

To determine the pathophysiological mechanism of procedural stroke, a correlation was made between the onset of neurological symptoms and available data on intra- and postprocedural haemodynamic complications, reported technical difficulties, and observed carotid patency. The topography of the procedural infarct (or haemorrhage) was assessed using available brain imaging.

Two investigators (AH and DC) analysed separately all available data described above to assess the likely procedural stroke mechanism according to the pre-defined following classification, in ischaemic stroke: (1) carotid-embolic, (2) haemodynamic, (3) thrombosis or occlusion of the carotid artery, (4) hyperperfusion, (5) cardio-embolic, (6) multiple, or (7) undetermined. In haemorrhagic stroke, it was determined whether stroke was related to: (1) hyperperfusion or (2) no hyperperfusion/ undetermined. For cases of uncertainty or disagreement, an adjudication meeting was held (AH, DC, FK, KRC-K, RLF, MMB, and TR) where the mechanism was defined by consensus or the mechanism was classified as unknown (Appendix I).

Stroke mechanism was considered as “carotid embolic” when there was angiographic evidence of an embolism or a clear association with shunt insertion and the onset of symptoms. Stroke mechanism was considered as “haemodynamic” when there was intra or postprocedural bradycardia (a heart rate less than 40 beats per minute), or asystole or any hypotension requiring treatment occurred. In the ICSS study protocol, no pre-defined cut-off blood pressure was used to define hypo- and hypertension. After the procedure, investigators were asked to provide information on the occurrence of hypo- and hypertension requiring treatment, but it was not mandatory to report actual blood pressure values. The presence of a subcortical borderzone infarct, defined as an infarct measuring more than 15 mm or multiple infarcts, regardless of size, in a Rosary-like pattern located (1) in the corona radiata or (2) located between superficial systems of the middle cerebral artery and anterior cerebral artery in the centrum semi-ovale area on the brain imaging performed after procedural stroke, was considered to be an indicator of a haemodynamic mechanism if the subcortical borderzone infarct was not present on baseline brain imaging. Where there was a haemodynamic infarct pattern, but absence of a haemodynamic complication, strokes were further assessed during the consensus meeting to confirm the haemodynamic mechanism. Stroke mechanism was considered to be “thrombosis or occlusion of the carotid artery” when the carotid artery was non-patent (defined as a ≥ 50% residual-stenosis or occlusion of the carotid) on imaging after procedural stroke or at re-exploration, irrespective of whether there was evidence for embolic or haemodynamic mechanism. Arrhythmia was defined as the development of a new arrhythmia detected in the procedural period. Stroke mechanism was determined as “cardio-embolic” when atrial fibrillation was detected on electrocardiogram immediately after stroke.

### Statistical analysis

A per-protocol analysis was performed including only patients randomised in ICSS in whom the allocated treatment was initiated as their first ipsilateral revascularisation procedure. Patients were excluded who received the alternative revascularisation procedure as their first treatment (cross-over) or who received no revascularisation treatment. Chi-square test was applied to compare frequencies of procedural stroke characteristics, haemodynamic events and technical difficulties between both treatment arms. A *p* value of <.05 was considered to be statistically significant.

## Results

### Patient flow

A total of 1,713 patients were enrolled in ICSS. For the present per-protocol analysis, 791 patients were included undergoing CAS and 819 undergoing CEA. A total of 85 patients suffered a procedural stroke, 58/791 (7.3%) patients assigned to the CAS arm and 27/819 (3.3%) patients assigned to CEA.

### Timing of stroke

**Figure 2:**
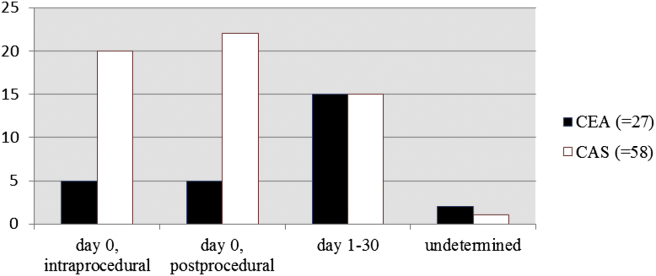
Timing of strokes following CEA or CAS (CEA = carotid endarterectomy; CAS = carotid artery stenting). The difference in timings of event between CEA and CAS was statistically significant (*p* = .014, chi-square test).

In the stenting arm, 43 of 58 strokes (74%) occurred on the day of the procedure (day 0). Twenty of the 58 (34%) were assessed as intraprocedural strokes. In one (2%) patient the onset of symptoms occurred on day 0, but it was not possible to determine the exact timing of stroke. In the surgery arm, 12 of 27 strokes (40%) occurred on the day of the procedure. Five of the 27 (19%) were considered intraprocedural, and for two (7%) strokes it was not possible to identify the exact timing of stroke (Fig. 2). Among patients assigned to the stenting arm, 15 of 58 strokes (26%) occurred between day 1 and day 30, on day 1 (2); 2; 3; 4; 5; 6; 7 (2); 8; 10; 11; 13; 23; and 26, respectively. In the surgery arm, 15 out of 27 strokes (56%) occurred between day 1 and day 30, on day 1 (4); 2 (2); 3 (3); 4; 5 (2); 6; 7; and 27, respectively.

### Severity of stroke

Overall, most strokes were classified as non-disabling (47/85 [55%]) (Table 1). Non-disabling strokes were more commonly seen in patients undergoing CAS (36/58 [62%]) compared with patients undergoing CEA (11/27 [41%]) (*p* = .066). At day 0 of CAS, 28 of 43 (65%) strokes were non-disabling. In the CEA arm, strokes on day 0 were non-disabling in five of 12 (42%) patients and disabling or fatal in seven of 12 (58%) patients (*p* = .143).

### Haemodynamic complications

Of the 55 patients in the CAS group, 18 (33%) developed haemodynamic disturbances: hypotension (*n* = 12), hypertension (*n* = 1), new arrhythmia (*n* = 2), and severe bradycardia (*n* = 2) (Table 2). One patient developed both hypotension and severe bradycardia. Among the 22 patients in the CEA group, 11 patients (50%) developed haemodynamic disturbances: hypertension (*n* = 6) and new arrhythmia (*n* = 5). There were significant differences between hypotension (*p* = .012), hypertension (*p* = .002), and new arrhythmia (*p* = .008) in the treatment arms. Among patients treated with CAS who developed hypotension, most (9 of 13 [69%]) were treated under local anaesthetics without sedation. All patients treated with CEA who developed hypertension were treated under general anaesthesia (6/6 [100%]).

### Technical difficulties

In eight patients, technical difficulties were encountered during the procedure, of which seven were in the CAS arm. In two patients either inability to advance the stent (1) or displacement of the stent (1) occurred. A slow flow or filling defect was noted immediately after post-stenting balloon dilatation, which correlated with the onset of neurological symptoms in three patients. One CAS procedure was considered to be technically difficult because of the presence of a Bovine arch configuration and a tortuous carotid artery, resulting in a long procedure time and the requirement of an additional stent. In the remaining patient the angioplasty balloon burst with extravasation of contrast and acute onset of neurological symptoms. In the CEA arm, one patient underwent a technically demanding procedure because of a high lesion with a prolonged clamping time. All eight strokes in procedures in which technical difficulties were encountered, were ischaemic, and most occurred on day 0 (7 of 8 [88%]) and were ipsilateral (7 of 8[88%]). The occurrence of technical difficulties did not differ significantly between both treatment arms *(p* = .406*)* (Table 2). For CAS patients intraprocedural control angiogram revealed a residual stenosis in seven out of 58 (12%) patients with procedural stroke, compared with 93 out of 709 (13%) patients without a procedural stroke (*p* = .820).

### Patency of the carotid artery on post-stroke imaging

Among 85 patients, 79 (93%) had brain imaging after the onset of the operative stroke. Post-stroke imaging of the carotid arteries was performed within 30 days in only 36 patients (27/58 CAS and 9/27 CEA) (Appendix II). In these 36 patients, patency was assessed by Duplex ultrasound (*n* = 22), CTA (8), selective angiography (3), MRA (2), or both US and CTA (1). Overall, in nine of 36 patients (25%; 6 CAS and 3 CEA) this revealed a > 50% residual stenosis and in seven of 36 patients (19%; 5 CAS and 2 CEA) an occlusion of the revascularised carotid artery. In the remaining 20 patients (56%; 16 CAS and 4 CEA) the carotid artery was found to be fully patent. Three patients (CEA arm) were reoperated at the onset of neurological symptoms. In two patients re-exploration confirmed a severe residual stenosis and outcome of stroke was fatal (1) and non-disabling (1). In one patient the carotid artery was found to be fully patent and clear of thrombus or intimal flap and outcome of stroke was non-disabling. Follow-up with duplex ultrasonography revealed a residual significant (>50%) stenosis in five of 85 patients (6%; 3 CAS and 2 CEA) at 1-month follow-up.

### Stroke mechanism

In 54 patients an obvious mechanism of stroke was determined. Based on the available information in the remaining 31 patients (22 CAS and 9 CEA), the adjudication committee defined the most likely mechanism in a further 12 patients at the consensus meeting. Therefore, overall, the mechanism of stroke was determined in 66 out of 85 patients (77%). In 19 patients the cause of stroke remained undetermined, but the timing of stroke could be determined in 18 of these 19 patients. Six strokes (32%; 4 CAS and 2 CEA) occurred intraprocedurally and 12 (64%; 10 CAS and 2 CEA) after the procedure. In the remaining patient, the timing of stroke was missing.

Of 66 patients (CAS 44 of 58 and CEA 22 of 27) with determined stroke mechanism, in the patients undergoing CAS, ischaemic stroke was caused by a haemodynamic mechanism (*n* = 15), carotid-embolism (10), or thrombosis or occlusion of the carotid (11). Other causes of stroke were hyperperfusion (2) or cardio-embolic (2). In three CAS patients multiple causes of stroke were identified: two patients with both a haemodynamic and carotid-embolic cause, and one patient with both thrombosis or occlusion of the carotid artery and a haemodynamic cause. In patients undergoing CEA, ischaemic stroke was caused by a haemodynamic mechanism (*n* = 5), followed by carotid-embolic (4), or thrombosis or occlusion of the carotid (4). Within this surgical treatment arm three ischaemic strokes were caused by a cardio-embolism and another two by hyperperfusion. There was no statistically significant difference in ischaemic stroke mechanism between both treatment arms *(p* = .479*)*. Among the seven haemorrhagic strokes, hyperperfusion was found more often in the CEA arm (*n* = 4) compared with the CAS arm (1). Overall, in 22 CEA patients with identified stroke mechanism, six (6/22 = 27%) had a postoperative stroke caused by hyperperfusion.

There was a significant difference in stroke mechanism between CAS and CEA for day 0 strokes occurring after the procedure (*p* = .022). Overall, a trend was seen towards an increased rate of carotid embolic and haemodynamic mechanism on day 0. For days 1–30, there was a trend towards an increased rate of thrombotic occlusion of the carotid (Table 3). For both ischaemic and haemorrhagic strokes there was no significant difference in stroke mechanism between non-disabling and disabling strokes (*p* = .383; *p* = .809).

The time interval between index event and procedure was ≤ 14 days in 17/85 (20%) patients. Patients treated within 2 weeks of the index event were more likely to develop procedural stroke caused by a haemodynamic mechanism compared with patients treated thereafter (8/17 [47%] versus 12/68 [18%]; *p* = .025).

## Discussion

The present study describes clinical characteristics and evaluates the cause of procedural strokes in symptomatic patients with atherosclerotic carotid stenosis, undergoing CAS or CEA in the International Carotid Stenting Study. This is the first detailed report of the most likely pathophysiological mechanism of procedural stroke comparing both treatment arms in patients treated either by CAS or CEA. In the CAS group most strokes occurred on the day of the procedure, were predominately minor, and most often caused by a haemodynamic mechanism. Strokes after CEA occurred more frequently in the postoperative phase, were predominately major, and most often caused by hyperperfusion.

Nearly all (97%) of the strokes associated with CAS were the result of infarction. In contrast, in the surgery arm a much larger proportion of patients (18%) suffered from a haemorrhagic stroke. Haemorrhagic strokes are considered a rare complication of carotid revascularisation but are generally more severe than ischaemic strokes.^12^ Most haemorrhagic strokes result from untreated postoperative hypertension leading to hyperperfusion injury, especially in patients with reestablishment of flow in previously infarcted cerebral tissue. Prolonged postoperative hypertension is one of the most important risk factors for haemorrhagic stroke after CEA.^13^ All haemorrhagic strokes in ICSS occurred several days after the procedure and most were preceded by severe hypertension. As a consequence, there is an opportunity for prevention of these strokes with strict postoperative blood pressure control.^14^ Given the delay in haemorrhagic stroke onset after revascularisation and the fact that hyperperfusion can complicate carotid revascularisation up to several weeks after the procedure, it is essential that the patient's blood pressure be well controlled before discharge. It might also be prudent to arrange for any patient in whom there has been concern about their blood pressure in the perioperative period to have their blood pressure checked again soon after discharge.

Nearly all strokes occurred in the ipsilateral hemisphere; however, a few (6) developed in a cerebral territory not directly related to the treated carotid artery. Non-ipsilateral strokes can be addressed to catheter-related disruption of the plaque in the aortic arch in patients undergoing CAS.^15^ The possible mechanism for non-ipsilateral strokes following CEA is not always clear. In the present cohort, three out of four non-ipsilateral strokes following CAS, occurred on the day of the procedure, suggesting a catheterisation-related mechanism. These findings are supported by the ICSS MRI substudy, in which patients treated with CAS more often had a new ischaemic DWI lesion on post-treatment scans.^16^

Timing of stroke relative to the postprocedural time interval is of clinical importance in terms of understanding the underlying mechanism of stroke. Therefore, a clear distinction was made between strokes that were apparent during the procedure or at awakening versus those strokes that occurred after a symptom-free interval. Cerebral deficit caused by carotid embolisation can occur intraprocedurally when atherothrombotic debris is released spontaneously in the unstable plaque or during manipulation of the carotid plaque (stent insertion, dissection phase, shunt insertion, and shunt dysfunction). Early postprocedural embolisation may be caused by embolism formation on the endarterectomised surface, a loose intimal flap, or originate from the external carotid artery. Very rarely, embolisation occurs in the late postprocedural period.^17^ In the present cohort, strokes caused by carotid embolism occurred in 85% on day 0. There was no significant difference between carotid embolic events in the treatment arms, although the absolute numbers of strokes were significantly greater on day 0 after CAS.

In the CAS arm, stroke was most often caused by a haemodynamic mechanism. Intraprocedural uncontrolled hypotension is a well-recognised and feared cause of cerebral deficit in patients undergoing carotid intervention.^18^ Haemodynamic depression is likely to result from manipulation of the carotid sinus and baroreceptor dysfunction.^19^ Other reasons for intraprocedural hypoperfusion are difficulty placing the shunt, prolonged clamping (CEA), or balloon dilation (CAS). In the present cohort, the proportion of haemodynamic strokes did not differ significantly between CAS and CEA, although the absolute numbers were again higher in the CAS arm. This is in keeping with earlier observations showing that the decrease in blood pressure in the first days after carotid intervention is greater in patients treated with CAS than CEA.^20^ Strict control of blood pressure also seems to be necessary during the weeks after CEA to avoid reperfusion syndrome.

Thrombosis or occlusion of the carotid artery has been described as a common cause of early postprocedural stroke after carotid intervention.^21^ Most thrombotic occlusions in the early postprocedural period have been suggested to be caused by technical errors during intervention and therefore can be corrected with re-establishment of cerebral perfusion.^22^ In the present cohort, patency of the carotid artery was assessed in only 36 of 85 patients, which revealed a residual stenosis or occlusion in 44% (16 of 36). It is possible that some of the patients in whom no post-stroke imaging of the carotid was performed might have benefitted from re-intervention.

This study has several limitations. First of all, because of its retrospective character it was not possible to identify the exact timing of stroke in three patients. Secondly, in ICSS no standard neurological assessment by a stroke physician or neurologist was performed until 1-month post revascularisation, although patients were seen by physicians earlier than 1 month post revascularisation if they suffered from a procedural stroke. As a consequence, minor signs and symptoms of cerebral deficit could have been missed. Third, in 19 of 85 patients, it was not possible to conclude on the underlying mechanism of stoke. Therefore, within this relatively small study population, caution must be applied to the validity of the proposed stroke mechanism in the remaining 66 cases. However, most of these strokes occurred on day 0, reaffirming the importance of thorough technique and close patient monitoring during the procedure and in the early postprocedural period. Fourth, in this post-hoc analysis the interpretation of available study data was necessary to define the most likely mechanism of stroke. Finally, analysis on the occurrence of hypo- and hypertension was based on the judgement of the physician that these events required treatment. Therefore, it was not possible to make a firm recommendation on threshold values for peri-procedural blood pressure. Additional studies are required to establish guidelines for the management of haemodynamic instability in the peri-procedural period. Further, in future studies and to allow comparison and pooling of data, it will be important to score procedural strokes according to the same method used in the present study.

## Conclusion

The mechanism of stroke following carotid intervention is diverse and haemodynamic disturbance is an important mechanism both in CAS and CEA. Blood pressure control requires careful attention in the peri-procedural period, which can potentially lower the incidence of procedural stroke. Further knowledge on the role of hypotension during stenting and the role of hypertension following surgery is required to further reduce procedural-related events of carotid revascularisation.

## Conflict of Interest

None.

## Funding

ICSS was funded by the UK Medical Research Council (MRC, grant number G0300411) and managed by the UK National Institute for Health Research (NIHR) on behalf of the MRC-NIHR partnership. The views and opinions expressed herein are those of the authors and do not necessarily reflect those of the NIHR Health Services Research programme of the Department of Health. Additional funding was supplied by grants from the Stroke Association (grant number TSA 2005/01 and TSA 2007/12), Sanofi-Synthelabo and the European Union. MMB's Chair in Stroke Medicine is supported by the Reta Lila Weston Trust for Medical Research. RLF was supported by the grant from the Medical Research Council. This work was undertaken at University College London, which received a proportion of funding from the UK Department of Health's National Institute for Health Research Biomedical Research Centres funding scheme.

## Figures and Tables

**Table 1:** Number (%) of patients with various procedural stroke characteristics among patients.

	CAS (*N* = 58)	CEA (*N* = 27)	*p*^b^
Stroke type			
Ischaemic^a^	56 (97)	21 (78)	.016
Haemorrhagic	2 (3)	5 (18)	
Unknown		1 (4)	
Arterial territory			
Ipsilateral	52 (90)	25 (93)	.965
Contralateral/vertebrobasilar	4 (7)	2 (7)	
Unknown	2 (3)		
Severity			
Non-disabling	36 (62)	11 (41)	.066
Fatal or disabling	22 (38)	16 (59)	

CAS = carotid artery stenting; CEA = carotid endarterectomy.

a Two patients, both treated with CAS, had a retinal infarction and were included in this category.

b *p* derived by use of the chi-square test comparing stroke type, territory, and severity between CAS and CEA.

**Table 2:** Haemodynamic and technical difficulties among patients with procedural stroke.

	Total (*n* = 77)^a^	CAS (*n* = 55) (intra vs. after the procedure)	CEA (*n* = 22) (intra vs. after the procedure)	*p*^b^
Haemodynamics				
- Hypertension	7	1 (0 vs. 1)	6 (1 vs. 5)	.002
- Hypotension	13^c^	13 (10 vs. 3)	0 (0 vs. 0)	.012
- New arrythmia	7	2 (1 vs. 1)	5 (2 vs. 3)	.008
- Severe bradycardia	3	3 (3 vs. 0)	0 (0 vs. 0)	.264
- No HD change	48	37	11	.158
Technical difficulties	8	7 (5 vs. 2)	1 (1 vs. 0)	.406

CAS = carotid artery stenting; CEA = carotid endarterectomy; HD = haemodynamic.

a Patients with missing data (8) were excluded from the analysis.

b One patient had both hypotension and severe bradycardia.

c *p* derived by use of chi-square test comparing haemodynamic events and technical difficulties between CAS and CEA.

**Table 3:** Stroke mechanism according to the procedural time interval.^a^

	Day 0, intraprocedural (*n* = 25)		Day 0, after the procedure (*n* = 27)		Days 1–30 (*n* = 23)	
	CAS	CEA	CAS	CEA	CAS	CEA
	*n* = 20	*n* = 5	*n* = 22	*n* = 5	*n* = 13	*n* = 10
Ischaemic						
- Carotid-embolic	4 (20)	0 (0)	5 (23)	2 (40)	0 (0)	2 (20)
- Haemodynamic	6 (30)	3 (60)	8 (36)	0 (0)	1 (8)	2 (20)
- Thrombosis or occlusion of the carotid artery	2 (10)	0 (0)	2 (9)	1 (20)	7 (54)	2 (20)
- Hyperperfusion	1 (5)	0 (0)	1 (5)	0 (0)	0 (0)	2 (20)
- Cardio-embolic	1 (5)	0 (0)	0 (0)	2 (40)	1 (8)	1 (10)
- Multiple	2 (10)	0 (0)	0 (0)	0 (0)	1 (8)	0 (0)
- Undetermined	4 (20)	2 (40)	6 (27)	0 (0)	3 (23)	1 (10)
	*p* = .654^b^		*p* = .022		*p* = .181	

	Day 0, intraprocedural (*n* = 0)		Day 0, after the procedure (*n* = 0)		Days 1–30 (*n* = 7)	
	CAS	CEA	CAS	CEA	CAS	CEA
	*n* = 0	*n* = 0	*n* = 0	*n* = 0	*n* = 2	*n* = 5
Haemorrhagic						
- Hyperperfusion	0 (0)	0 (0)	0 (0)	0 (0)	1 (50)	4 (80)
- No hyperperfusion/undetermined	0 (0)	0 (0)	0 (0)	0 (0)	1 (50)	1 (20)
					*p* = .427	

CAS = carotid artery stenting; CEA = carotid endarterectomy.

a The three strokes with unknown timing are not included in the table. In these cases the mechanism was carotid-embolic (1), thrombosis or occlusion of the carotid artery (1), and undetermined (1).

b *p* derived by use of chi-square test comparing stroke mechanism (on, respectively, day 0, intraprocedural, day 0 after the procedure, and days 1–30) between CAS and CEA.
