# Supplementary material for: Mechanism of Procedural Stroke Following Carotid Endarterectomy or Carotid Artery Stenting Within the International Carotid Stenting Study (ICSS) Randomised Trial
Source: Eur J Vasc Endovasc Surg. 2015 Sep;50(3):281–8. doi: 10.1016/j.ejvs.2015.05.017 (PMC4580136; doi:10.1016/j.ejvs.2015.05.017)
Supplement: Supplementary file 1 [file mmc1.doc]

**Appendix I.** Flow chart of stroke mechanism classification

Procedural strokes

Ischaemic

Haemorrhagic

Clear mechanism

Unclear mechanism

Clear mechanism

Unclear mechanism

Consensus meeting

Consensus meeting

(1) carotid-embolic

(2) haemodynamic

(3) thrombosis or occlusion of the carotid artery

(4) hyperperfusion

(5) cardio-embolic

(6) multiple

(7) undetermined

(1) hyperperfusion

(2) no hyperperfusion/ undetermined

Separate analysis of stroke mechanism by two independent investigators

**Appendix II. Patency of the carotid among patients with procedural strokes**

|  | CAS  (*n*=27) | CEA  (*n*=9) | *p* ^a^ |
| --- | --- | --- | --- |
| Patent carotid | 16 (59) | 4 (44) | .598 |
| > 50% residual stenosis | 6 (22) | 3 (33) |
| Carotid occlusion | 5 (19) | 2 (22) |

CEA = carotid endarterectomy; CAS = carotid artery stenting.

^a^ *p* derived by use of chi-square test comparing patency of the carotid artery between CAS and CEA.
